# *Trichoderma harzianum* in Biocontrol of Maize Fungal Diseases and Relevant Mycotoxins: From the Laboratory to the Field

**DOI:** 10.3390/jof11060416

**Published:** 2025-05-27

**Authors:** Ivana Mitrović, Petar Čanak, Sonja Tančić Živanov, Hunor Farkaš, Marko Vasiljević, Svetlana Ćujić, Miroslav Zorić, Bojan Mitrović

**Affiliations:** 1 Faculty of Technology Novi Sad, University of Novi Sad, Bulevar cara Lazara 1, 21000 Novi Sad, Serbia; 2 Maize Research Institute Zemun Polje, Slobodana Bajića 1, 11185 Belgrade, Serbia; pcanak@mrizp.rs (P.Č.); bmitrovic@mrizp.rs (B.M.); 3 Institute of Field and Vegetable Crops, Maksima Gorkog 30, 21000 Novi Sad, Serbia; sonja.tancic@ifvcns.ns.ac.rs; 4 Patent Co., doo, Vlade Ćetkovića 1a, 24211 Mišićevo, Serbia; hunor.farkas@patent-co.com (H.F.); marko.vasiljevic@patent-co.com (M.V.); svetlana.cujic@patent-co.com (S.Ć.); 5 LoginEKO doo, Tiski red 117, 23207 Aradac, Serbia; crop.biometrics@gmail.com

**Keywords:** *Trichoderma harzianum*, bioprocess, biological control, maize ear disease, mycotoxins, *Fusarium graminearum*, *Aspergillus flavus*

## Abstract

**Background:** Maize, one of the world’s most important food and feed crops, is often threatened by fungal infections that not only reduce yields but also contaminate grains with harmful mycotoxins. **Methods:** This study evaluated the biocontrol potential of *Trichoderma harzianum* K179 as an eco-friendly alternative to synthetic fungicides for protecting maize from two major pathogens, *Fusarium graminearum* and *Aspergillus flavus*. *T. harzianum* K179 was cultivated in a lab-scale bioreactor, and its antifungal activity was assessed through in vitro inhibition assays and two-year field trials. During the field trial, maize ear disease severity, yield, and mycotoxin levels in maize samples were monitored to assess the efficacy of the produced *Trichoderma* biopreparation. **Results:** In laboratory tests, *T. harzianum* K179 significantly inhibited both target pathogens. Field trials demonstrated that seed treatments with the *Trichoderma* bioagent reduced ear rot severity and increased grain yield compared to untreated and chemically treated controls. Notably, maize samples from *T. harzianum*-treated plots contained lower concentrations of key mycotoxins, including fumonisins and aflatoxins. **Conclusions:** These findings highlight the usefulness of *T. harzianum* K179 in integrated pest management strategies, offering a sustainable solution that enhances crop safety and productivity while mitigating the environmental risks associated with chemical fungicides.

## 1. Introduction

As a final product of the plant life cycle, seed plays an important role in reproduction, survival, renewal of species, but also in the transmission of infection [1]. The seed of cultivated plants represents a suitable substrate for the development of microorganisms and pathogens, among which the most important place is occupied by fungi. Pathogenic microorganisms, primarily fungi that colonize maize seed can reduce grain yield and seed quality. Fungal infections in maize can lead to smaller kernel size, reduced protein content, and adverse effects on germination, resulting in lowered yield and feed quality [2]. *Fusarium* and *Aspergillus* species are among the most important causes of maize diseases. The most common *Fusarium* species detected on maize in Europe are *F. graminearum* and *F. verticillioides* [3,4,5,6]. Yield losses due to *Fusarium* ear rot on maize in Europe can range from 10% to 30% on average, but in severe cases, particularly under conditions of high infection, yield reductions can exceed 40% [7,8].

On the other hand, *Aspergillus* species, particularly *Aspergillus flavus* and *A. parasiticus*, are known to cause aflatoxicosis in maize and are predominant in warmer, tropical, and subtropical climates [9]. Recently, due to climate change their prevalence has shifted to temperate regions as well. Regions with warm, dry conditions favor the growth of *Aspergillus* species, making southern Europe particularly vulnerable, so that maize crops in countries like Spain, Italy, and southern France are often exposed to such conditions. However, the occurrence of *Aspergillus* infections has also been reported in other European regions, with the presence of *A. flavus* confirmed in Portugal, Western Romania, Serbia, Croatia, Slovenia, and Hungary [5,10,11]. Furthermore, the species *A. parasiticus* was isolated from maize seed for the first time in the growing season of 2012 in Serbia [12,13], while occurrence of *A. niger* and *A. flavus* on maize in Serbia was detected in the period 2008–2012 by Lević et al. (2013) [14]. On average, maize yield losses from *Aspergillus* infections in Europe range from 5% to 20%. However, in severe cases or during high-infection years, losses can be much higher, potentially exceeding 30% [15,16].

In addition to their pathogenicity, these species are known to have high toxicity potential due to the production of different secondary metabolites—mycotoxins [17]. The most important mycotoxins produced by *Fusarium* species are trichothecenes (T2, HT-2), zearalenone (ZEN), and fumonisins (FUM) [18]. On the other hand, aflatoxins are the most toxic secondary metabolites produced by *A. flavus* [19] and *A. parasiticus* [20], while ochratoxins are a group of mycotoxins produced mainly by *A. ochraceus* and *A. carbonarius*, but also by *A. niger* [21].

Produced mycotoxins reach an animal’s body through feed containing infected maize and lead to various negative changes. However, by consuming the meat, milk or eggs of infected animals, humans ingest mycotoxins that can cause various consequences in the human body [17]. *F. graminearum* is known to produce two significant toxins: trichothecenes deoxynivalenol (DON) and estrogenic ZEN. Acute DON intake is most often manifested by characteristic toxicological effects, such as reduced food intake and vomiting in animals [22]. Trichothecenes have a significant effect on protein synthesis and DNA and RNA synthesis which indicates the seriousness of their consumption [23]. On the other hand, ZEN affects the reproductive system in animals (hemorrhaging and atrophy of the ovaries) [22].

Another important *Fusarium* species, *F. verticillioides*, produces FUM, of which the most toxic is fumonisin B1 (FB1), which shows nephrotoxic and hepatotoxic effects on experimental animals [24]. This mycotoxin mainly causes abdominal pain and diarrhea in humans, but there is also information about the esophageal cancer [25]. The International Agency for Research on Cancer characterized FB1 as a group 2b possible carcinogen for humans [24]. Due to the harmful effects of FUM, The European Union (EU) established a recommendation of 1 µg FUM/kg body weight per day as the acceptable daily consumption for people [26].

According to the World Health Organization (WHO) several types of aflatoxins (AFs) occur in nature, but four aflatoxins, B1, B2, G1 and G2, are particularly dangerous to humans and animals. Large doses of aflatoxins lead to acute aflatoxicosis that can be life-threatening, causing liver damage. Chronic aflatoxin poisoning results in immunosuppressive and carcinogenic changes [27]. According to International agency for Research on Cancer aflatoxin B1 (AFB1) is classified in group 1a carcinogenic compounds for humans [28]. The highest standard level is set by the EU, where total AFs and AFB1 cannot exceed 4 µg/kg and 2 µg/kg, respectively [29].

Given that maize is a world-important agricultural crop used primarily in the diet of animals, adults, and babies as well, the presence of mycotoxins and fungal diseases in maize must be controlled. Climate change at the global level has proven to be the cause of numerous problems in the field and one of the leading ones is related to the appearance of mycotoxins in crops [30]. Due to the impossibility of controlling climate change, the application of fungicides has become the most common reliable solution for crop protection against pathogenic fungi and their dangerous toxins. However, the use of synthetic fungicides has led to another problem, which is their harmful effects on non-target organisms, human health, and the environment [31,32].

Because of all the above, scientists around the world have focused their research on finding more sustainable and eco-friendly ways for plant disease control and crop production. Therefore, the notion of biocontrol is closely linked to sustainable agricultural practices that are necessary to protect the environment and agricultural resources [32]. While fungicides have a temporary effect and mostly require reapplication, biocontrol agents have the ability to bind to the ecosystem in terms of reproduction and colonization of the rhizosphere. Fungi that belong to genus *Trichoderma* fully colonizes roots as they grow and provides at least season-long benefits to plants [33], producing antifungal compounds and enzymes that inhibit the growth of soil-borne pathogens, promoting plant health and thus reducing the need for chemical pesticides [34,35,36]. *Trichoderma* has proven to be very successful in biocontrol [32,37] inhibiting the growth of various phytopathogens [38,39], but also as a plant growth promoter of various crops—maize, wheat, soybean, lettuce, tomato, pepper, chilli, etc. [40,41,42,43,44]. The fact that *Trichoderma* species are resistant to most chemical pesticides makes those species good candidates for both, biocontrol and integrated control [35,45]. Worldwide distribution, fast growth and high spore production make those species easy to isolate, but not all *Trichoderma* strains are effective, so strain selection is of crucial importance.

Seed treatment is an effective strategy for controlling seed-borne diseases, with various methods available to suit different agricultural needs [46]. Due to the recognized need for seed treatment in the 20th century, specialized devices for seed treatment were developed with capacities that enabled their industrial application. These machines were developed for the purpose of applying preparations to seeds before sowing, thus contributing to the protection of agricultural crops from the beginning of their life cycle.

Accordingly, this study aimed to produce the *Trichoderma harzianum* K179 biocontrol agent in a lab-scale bioreactor and evaluate its antagonistic effect against major maize pathogens under in vitro and field conditions. Key parameters, including maize ear disease severity, yield, and mycotoxin levels in maize samples, were monitored to assess the efficacy of the produced *Trichoderma* biopreparation.

## 2. Materials and Methods

### 2.1. Fungal Material

All fungal strains used in the study were obtained from the Institute of Field and Vegetable Crops, Novi Sad (IFVCNS), Serbia, where they had been isolated according to the standard procedure of the IFVCNS Laboratory for Phytopathology, purified to single-spore strains according to Leslie and Summerell (2006) [47], identified and collected. All strains used in the study were stored on PDA slants in a refrigerator at 4 °C until use, with regular revitalization every 6 months.

*T. harzianum* strain K179 was obtained from soil sample collected at Belegiš, Vojvodina Province, Serbia. It was first identified according to its morphological characteristics following Samuels and Hebbar (2015) [48], which was confirmed by molecular identification through ITS1 and ITS2 regions, separated by the 5.8S ribosomal RNA gene (ITS, Internal Transcribed Spacer)—NCBI Acc. No. MN448464. A PCR assay for the translation elongation factor (TEF-1a) gene was conducted with primers TEF1-728 F and TEF1-986R, and the partial sequence obtained showed 99% identity with sequences of *T. harzianum* (KY236120.1; KY236119.1; KT357557.1) in the NCBI database and 99% identity with *T. harzianum* (ISTH-42GJS97-263) in the TrichoBlast ISTH database. *T. harzianum* K179 was used for in vitro and the field experiments, and inoculum preparation was performed according to Mitrović et al., 2023 [49]. Obtained inoculum was further used in a laboratory bioreactor experiment.

*F. graminearum* K28 and *A. flavus* K328 were isolated from infected maize seed grown at Rimski Šančevi, Vojvodina Province, Serbia. Morphological identification of single-spore strains K28 and K328 was achieved by following Leslie and Summerell (2006) [47] and Klich (2002) [50] identification manuals and by comparing macroscopic and microscopic characteristics of strains with the reference strain of *F. graminearum* sensu stricto (NRRL 5883), originating from maize in Ohio (USA), and the reference strain of *A. flavus* CBS 100927A, respectively. *F. graminearum* K28 and *A. flavus* K328 strains, as representatives of the most important maize pathogens, were used as test pathogens in vitro biocontrol study that was carried out according to Mitrović et al. (2023) [49].

### 2.2. Bioreactor Experiment

The experiment in a lab-scale bioreactor with a total volume of 3 L was carried out using an aeration intensity of 1.5 vvm and a stirring speed of 250 rpm as previously described in the study by Mitrović et al. (2023) [49]. The temperature was maintained constant at 28 °C during the bioprocess. The initial pH of the medium was about 6 before sterilization and was not adjusted afterwards. The bioprocess lasted until a cell concentration of 10^7^ CFU/mL was reached. Appropriate electrodes and sensors, which are an integral part of the applied laboratory bioreactor equipped with the appropriate software package BioPAT® MFCS/win 3.1 (Biostat, Göttingen Germany), were used for bioprocess monitoring. The medium used for the cultivation of *T. harzianum* K179 had the following composition (g/L): dextrose (10), soybean flour (6.87), K_2_HPO_4_ (1.51), KCl (0.5) and MgSO_4_ × 7H_2_O (0.5). Before starting the bioprocess, 200 mL of the previously prepared *T. harzianum* K179 inoculum was added to 2 L of medium for cultivation.

Cultivation broth was sampled every 3 h of bioprocess in order to monitor the concentration of multiplied *T. harzianum* K179 cells by using an automated cell counter (Countess, ThermoFisher, Waltham, MA, USA). The bioprocess was stopped when the result of three simultaneous measurements showed a cell concentration of 10^7^ CFU/mL.

### 2.3. In Vitro Experiment

For the in vitro experiment, 10 mL of the produced cultivation broth was set aside in order to confirm its biocontrol/antagonistic activity against phytopathogens. The rest of the cultivation broth was used for further field experiments. In vitro biocontrol test of the produced *T. harzianum* K179 bioagent was performed in Ø 90 mm Petri dishes. As representative phytopathogens, *F. graminearum* K28 and *A. flavus* K328 strains were used. The test was performed by well diffusion method described by Grahovac et al. (2020) [51]. The second layer, consisting of 1.2% PDA and pathogen, was applied to the first layer of pure PDA medium. The pathogen was previously prepared in PDB medium, filtered through a double layer of sterile gauze, and added to the previously prepared 1.2% PDA medium at a concentration of 35%. After solidification of the abovementioned media, 100 μL of the tested *T. harzianum* 179 cultivation broth was added to the prepared wells. After 7 days of incubation at 27 °C, the diameters of inhibition zones formed against phytopathogenic strains were measured. The in vitro biocontrol experiment was performed in triplicate.

### 2.4. The Field Trial

The field experiment was carried out at the site Rimski Šančevi (45° 46′ north latitude and 19° 20′ east longitude, Novi Sad, Serbia) during the 2021 and 2022 growing seasons (May–October) according to the standard protocol. The field where the experiment was conducted, is located in the northern part of Serbia, an area with a semi-arid continental climate. Data on climate conditions during the analyzed seasons are publicly available at the Republic Hydrometeorological Service of Serbia and in the Supplementary Materials. Commercial hybrid NS2662 intended for animal and human consumption was used in the study. In order to avoid artificial inoculation with phytopathogens in the field, maize seeds that were naturally infected with *Aspergillus* and *Fusarium* pathogens were selected and used for a field experiment. Preliminary analysis of seed fungal infection according to the ISTA procedure was done by Accredited Laboratory for seed testing within the IFVCNS. The results showed that seed of hybrid NS2662 intended for the field test was infected with fungi from the genera *Aspergillus* spp. (2%) and *Fusarium* spp. (4%).

The field experiments were set up as a split-plot design including four treatments and a control, all distributed in four blocks. The experimental plot area per treatment was 19.5 m^2^. The plot included four rows, each measuring 6.5 m, with 0.75 m between rows and 0.2 m between plants in row. Standard cultivation practices were applied according to the local agroecological conditions.

**Figure 1:**
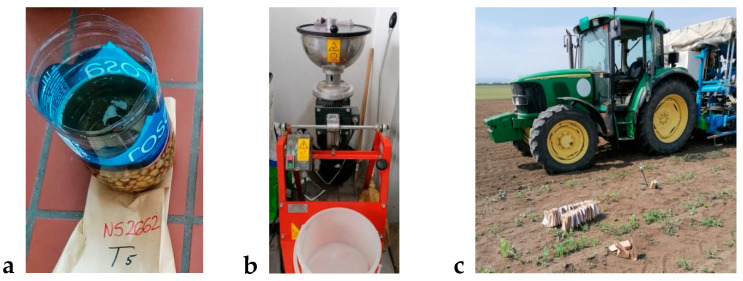
Immersion treatment (**a**), treatment with a coating machine (**b**), and sowing (**c**).

Produced *T. harzianum* K179 cultivation broth used for the field experiment contained 10^7^ CFU/mL (Countess, ThermoFisher). Seed treatments before sowing were as follows: T1—control (untreated seed); T2—synthetic fungicide (25 g/L fludioxonil + 10 g/L metalaxyl-m); T3—*T. harzianum* K179 cultivation broth applied using a seed-coating machine; T4—*T. harzianum* K179 cultivation broth applied using a seed-coating machine with the addition of glue; and T5—immersion in *T. harzianum* K179 cultivation broth for 1 h. Figure 1a,b shows the method of seed treatment before sowing and the sowing procedure (Figure 1c).

The field bioefficiency parameters that were measured/monitored were disease severity and grain yield.

Disease severity was determined on each tested maize ear using a 7-point scale in relation to ear infection, where 1 = 0% infection; 2 = 1–3% infection; 3 = 4–10% infection; 4 = 11–25% infection; 5 = 26–50% infection; 6 = 51–75% infection; and 7 = 76–100% infection [52]. Twenty maize ears harvested sequentially from the side rows of each experimental plot were used for evaluation.

Grain yield was estimated after harvest. The two middle rows of each experimental plot were used to estimate the grain yield [53]. The experiment was sown and harvested by machines. At harvest, grain weight and moisture content were recorded for each experimental plot. Grain yield (t/ha at 14% moisture) was determined by the following formula:

weight of sample (kg) × (100 − moisture content %)/86 × (10,000/plot area)

### 2.5. Mycotoxins Detection

Both side rows of each treatment were harvested, and the collected maize kernels were adequately homogenized. After homogenization, maize kernels of each applied treatment in four repetitions were delivered in standard bags for mycotoxins detection. The delivered samples were passed through a separator and 500 g of a representative sample was used for analysis. Kernel mycotoxin analysis was conducted for two spontaneously selected repetitions of the same treatment.

RETSCH ZM200 mill was used to homogenize the samples. Following the manufacturer’s recommendations, the mill was cleaned after each sample to prevent sample cross-contamination. Sample extraction was performed using 20 mL of extraction solution (acetonitrile:water:formic acid = 79:20:1). Samples were further placed on a horizontal shaker (MAXQ 4450, ThermoScientific) for 90 min (250 rpm). After 90 min, the samples were centrifuged (Eppendorf centrifuge 5804) for 5 min at 4500 rpm. The extracts were diluted with ultra-pure water (<0.055 µS/cm). Before analysis, the liquid samples were passed through a nylon filter (0.22 µm; AMTAST, Lakeland, FL, USA, p/n: SFNY013022NA).

The analysis was performed on an LC-MS/MS Agilent Technologies (Santa Clara, CA, USA) device equipped with the following: Agilent ZORBAX Rapid Resolution HT 4.6*50 mm 1.8 µm column, ZORBAX Eclipse Plus C18, 2.1 mm, 1.8 µm, UHPLC guard column, autosampler Agilent Technologies 1290 series II with thermostat and column oven, Agilent Technologies 1290 series II Flex pump and MS/MS detector Agilent Technologies 6460c MS/MS QQQ with Jet Stream electrospray ion source. The mass spectrometer analyses were carried out using selected reaction monitoring channels in positive electrospray ionization (ESI+) mode.

Maize samples were analyzed to determine the potential presence of the following mycotoxins: aflatoxin B1 (AB1), aflatoxin B2 (AB2), aflatoxin G1 (AG1), aflatoxin G2 (AG2), deoxynivalenol (DON), fumonisin B1 (FB1), fumonisin B2 (FB2), T-2, HT-2, ochratoxin A (OTA), and zearalenone (ZEN).

Quantification of mycotoxins by LC-MS/MS was achieved by adding internal standards for each group of mycotoxins [13C17] aflatoxin B1; [13C15] deoxynivalenol; [13C18] zearalenone; [13C20] ochratoxin A; [13C34] aumonisin B1; [13C24]; T-2.

### 2.6. Data Analysis

The obtained values of inhibition zone diameters in the in vitro biocontrol experiment were processed in the program Statistica.Ink 13.0, using One-way ANOVA.

Grain yield was analyzed using a linear mixed-effects model with the following formulation (1):

(1) y=μ+blk+trt+ε

where y is the grain yield; μ is the overall mean; *blk* is the random effect of block with blk~N(0,σ_blk^2); *trt* is the fixed effect of the treatment; *ε* is the random residual effect, with ε~N(0,σ_ε^2). Two linear models with the same formulation were fitted on a per-year basis. The means of treatments were compared with a Tukey multiple comparison test. The results were graphically presented as means of treatment with their corresponding standard error of difference, providing a clear depiction of treatment differences.

The proportional odds model (POM) is a type of ordinal regression used to analyze outcomes that are naturally ordered-in this case, the disease severity ratings caused by phytopathogenic fungi. In our two-year experiment evaluating five biological treatments, the disease severity was assessed on an ordinal scale, and the POM was estimated separately for each year. In this model, instead of modeling the probability of each individual category directly, we model the cumulative probabilities (π). Specifically, if *Y* is the ordinal response variable with categories 0, 1, …, *J*, the model considers the cumulative probability γj=P(Y≤j) or each cut-point *j*. These cumulative probabilities are linked to the predictors (e.g., the type of biological treatment) using the logistic function (2):

(2) logit(γj)=log(γj/(1−γj))=θj−β

where *θj* represents the threshold (or cut point) for ordinal category *j*, *β* is the coefficient determining the effect of the biological treatments, and *X* denotes the independent variable (i.e., the treatment effect) in the POM. Additionally, the model is referenced to the control treatment, so that the estimated coefficients for the other treatments reflect their deviation from this reference, enabling direct comparisons of treatment effects. A key assumption of the POM is that the effect of the predictors (as captured by *β*) remains constant across the different cumulative logits. This is known as the proportional odds assumption, which implies that the odds ratio between any two adjacent categories is the same regardless of where the threshold is set [54].

Parameters in this model are typically estimated using maximum likelihood methods, and the significance of treatment effects is assessed. By modeling the cumulative probabilities, the POM provides an adequate framework to evaluate how different treatments shift the overall distribution of disease severity. The POM accommodates the ordinal nature of the disease severity ratings and allows for a direct comparison of the efficacy of the five biological treatments across the two separate experimental years.

## 3. Results

### 3.1. In Vitro Biocontrol Experiment

**Figure 2:**
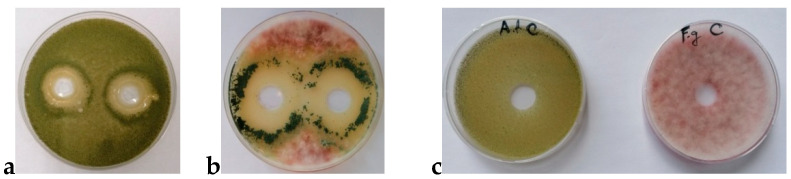
Antagonistic activity of *T. harzianum* K179 cultivation broth (10^7^ CFU/mL) against *A. flavus* (**a**) and *F. graminearum* (**b**); controls (**c**).

In order to test the efficiency of the produced *T. harzianum* K179 cultivation broth against isolates of *F. graminearum* K28 and *A. flavus* K328, the in vitro biocontrol experiment was performed. The results of the obtained inhibition zone diameters are shown in Figure 2.

The results show that the maximum mean diameters against both tested pathogens were formed after 36 h of *T. harzianum* 179 cultivation in the bioreactor. From Figure 2, it can be concluded that the pathogenic maize isolate, *F. graminearum* K28 was more sensitive to the produced *T. harzianum* K179 cultivation broth compared to isolate *A. flavus* K328. Maximum obtained mean diameter of the zone formed against isolate *F. graminearum* K28 was 57.67 mm, while the maximum mean diameter formed against isolate *A. flavus* K328 was 36.67 mm. Certainly, based on the obtained results, it can be concluded that the produced *T. harzianum* 179 cultivation broth shows extremely good antifungal activity in vitro against the tested maize phytopathogens. This result served as the basis for further testing of *T. harzianum* 179 cultivation broth under in vivo conditions in the field.

### 3.2. Field Experiment

#### 3.2.1. Disease Severity

The effectiveness of the applied treatments was estimated using the disease severity scale and the appropriate regression model, proportional odds model, for ordered categorical data. The results of the study showed that there was a statistically significant difference between the applied treatments at the significance level of *p* < 0.01 in terms of disease severity.

The results presented in Table 1 show that treatments T3, T4, and T5 (treatments with the applied *T. harzianum* K179 bioagent) showed statistically very significant differences compared to control (*p* < 0.01), while treatment with synthetic fungicide T2, showed marginally significant differences compared to control (*p* < 0.05) in 2021 season. This is in accordance with results from field experiments conducted by Sangeetha (2009) [55] and Degani and Dor (2021) [56].

**Figure 3:**
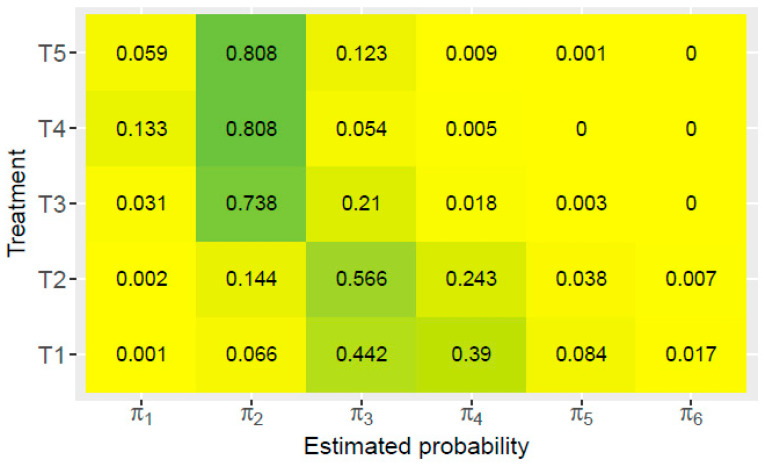
Cumulative probabilities of the proportional odds model for different treatments for hybrid NS 2662 from the POM (2021).

In order to analyze the obtained data using disease severity scale and in accordance with the applied model, the cumulative probability is presented in Figure 3. The severity of the disease was determined by grades from 1 to 7, while cumulative probabilities, π1–π6, were calculated accordingly. The probabilities for categories 5, 6, and 7 will be considered in more detail, as they indicate the highest severity of the disease and consequently the potentially highest yield loss [54].

Analysis of individual treatments shows differences in the probability of disease severity. During season 2021, the control treatment, T1 (Figure 3), had the highest estimated probability for grades 3 (0.442) and 4 (0.390), while, for the other grades, the cumulative probability was less than 0.1. The lowest probability for the T1 treatment was recorded for grade 1 (0.001), indicating the presence of a very small number of completely healthy maize ears. A similar cumulative probability was observed for the synthetic fungicide treatment T2. Namely, the highest estimated probability was registered for grade 3 (0.566), significantly lower probability for grade 4 (0.243) and 2 (0.144), while other disease severities had probabilities with values less than 0.1.

From Figure 3, it can be seen that for treatments T3, T4, and T5 the dominant cumulative probability was for grade 2. This indicates that by applying these treatments, the largest number of tested samples were almost healthy. Treatments T4 and T5 had the same cumulative probability value of 0.808 which was higher compared to treatment T3 (0.738). Also, all treatments with the applied *T. harzianum* K179 biocontrol agent (T3–T5) had a cumulative probability 0 for grade 6, while for T4 treatment cumulative probability 0 was also present for grade 5. This indicates that treatment with the applied *T. harzianum* K179 had no maize ears rated with scale 7. Compared to the other treatments, the T4 treatment also had the highest estimated probability for grade 1 (0.133) indicating that in the trial conducted during the 2021 season, the T4 treatment provided the highest number of completely healthy maize ears.

In general, compared with the results of the control treatment (T1), it can be concluded that the highest estimated probabilities for grades 1 and 2 were observed in treatments T4 and T5, i.e., treatments in which the *T. harzianum* K179 biocontrol agent was used. Therefore, in those treatments, the highest percentage of healthy maize ears was registered. The reason for this result was probably the fact that, in these treatments, *T. harzianum* K179 better colonizes the root of the plant and leads to the induced systemic resistance of the plant against pathogenic fungi [57].

It can be assumed that the difference between treatments T3 and T4, in which the *T. harzianum* K179 cultivation broth was applied using a seed-coating machine, was due to the application of glue in the treatment T4. On the other hand, both treatments T4 and T5 showed good antifungal activity. Specifically, in treatment T4, the glue enabled better fixation of the biocontrol agent *T. harzianum* K179 to the seeds, while in treatment T5, the seed immersion in *T. harzianum* K179 broth for 1 h enabled partial soaking of the seeds and thus better adhesion of *T. harzianum* K179 to the seed.

Further, Table 2 shows the results of the field trial conducted during 2022 at the same location and using the same experimental design. Observing the results obtained during the 2022 growing season (Table 2), it can be noticed that there was a statistically very significant difference (*p* < 0.01) between the control treatment T1 and treatments T3, T4 and T5. In contrast to the previous year (Table 1), in the field experiment conducted in 2022, the treatment with the synthetic fungicide (T2) was not statistically significant in relation to the control treatment. Thus, the results indicate the great potential of *T. harzianum* K179 as a biocontrol agent for the most common maize pathogens.

**Figure 4:**
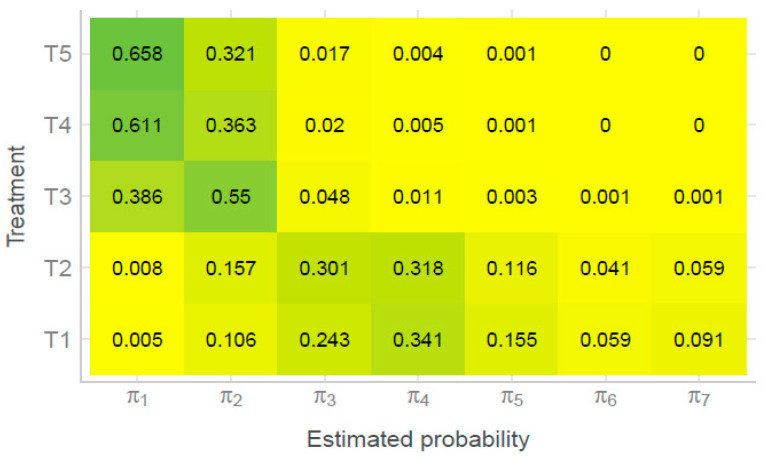
Cumulative probabilities of the proportional odds model for different treatments for hybrid NS 2662 from the POM (2022).

In order to analyze the obtained data for disease severity in accordance with the applied model, the cumulative probability for the 2022 season is presented in Figure 4. The severity of the disease was determined by grades from 1 to 7, while cumulative probabilities, π1–π7, were calculated accordingly.

From Figure 4, it can be observed that treatments T4 and T5 had the highest estimated probability for grade 1. This indicates that these treatments produced the healthiest maize ears, with no visible disease symptoms. Additionally, both treatments showed a cumulative probability of zero for categories 6 and 7 on the ordinal disease severity scale, indicating that no ears exhibited infection levels above 51%.

The T3 treatment also showed statistically significant differences compared to the control treatment. In the T3 treatment, the highest cumulative probability was for category 2, suggesting that this treatment also has as a biological control treatment.

On the other hand, no statistically significant difference was observed between the treatments T1 and T2 (Table 2). Both treatments had the highest value of cumulative probability for grade 4, indicating that most maize ears exhibited severe signs of infection. At the same time, the lowest values of cumulative probability were recorded for grade 1, indicating that the least number of completely healthy maize ears was recorded in treatments T1 and T2 (Figure 4).

#### 3.2.2. Grain Yield

**Figure 5:**
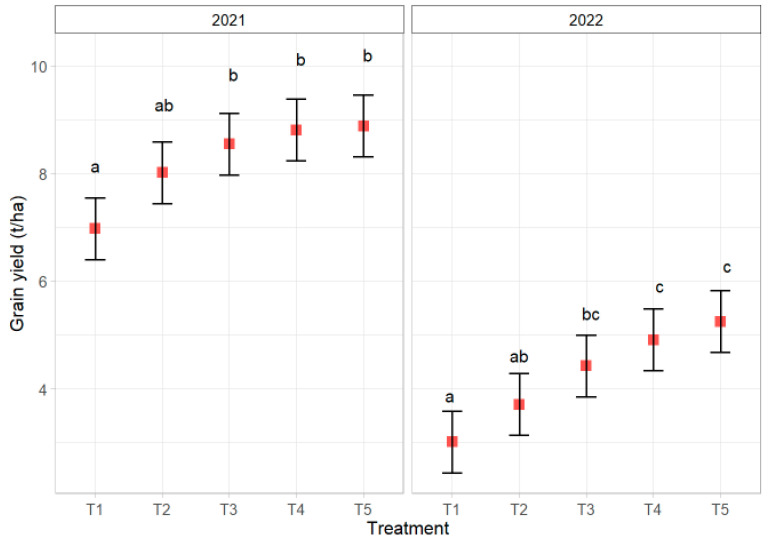
Grain yield for hybrid NS 2662 during the 2021 and 2022 growing seasons. Values in the same year followed by the same letter are not statistically significantly different at the 0.05 level.

Grain yield is a key indicator of the effectiveness of seed treatments on crop production success. Figure 5 presents maize yield results during the 2021 and 2022 growing seasons. From Figure 5, it can be concluded that there were significant differences in yield between the two growing seasons. It is obvious that the influence of climatic conditions, which mostly vary from year to year, can contribute to an increase/decrease in yield. In addition to external influences, certainly, the influence of crop genotype also plays a significant role [58].

If the results for the two seasons are compared, it can be concluded that the yield is higher in 2021 compared to the 2022 growing season. The reason for this could be the aforementioned climatic conditions, which in 2021 were more favorable for maize cultivation. On the other hand, if we compare the effect of the treatment on the yield, it can be registered that there are significant statistical differences between treatments. Therefore, in both seasons, the control was at the same level of statistical significance (marked with a lowercase letter a) as the treatment T2—synthetic fungicide, while the treatments with the applied *T. harzianum* K179 showed a significantly better yield compared to the control.

During the 2021 season, it was observed that the treatments T2, T3, T4, and T5 are also at the same level of statistical significance (indicated by a lowercase letter b) in terms of grain yield. However, considering the less favorable season for maize cultivation, the 2022 season, the conclusion is different. Namely, treatments T4 and T5 (indicated with a lowercase letter c), i.e., treatments containing *T. harzianum* K179, were at the highest level of statistical significance compared to control and synthetic fungicide. Therefore, treatments with applied *T. harzianum* K179 showed better grain yield compared to the control T1, but at the same time compared to the treatment T2 with a synthetic fungicide.

### 3.3. Mycotoxins

In order to obtain all the necessary information about the impact of different treatments during two different growing seasons, an analysis of the presence of mycotoxins in maize was performed. Table 3 contains the results obtained from the analysis of maize samples collected in October 2021. The results showed that in all samples, regardless of the applied treatment, the presence of aflatoxin, ochratoxin A, ZEN, and DON was not found. The reason for this may be the influence of favorable climatic factors during the 2021 season, which was favorable for maize cultivation [59] and at the same time unfavorable for the development of fungal infections [60].

In addition to the above, the presence of fumonisins B1 and B2, as well as the trichothecene mycotoxins T-2 and HT-2, was detected in the samples. Analyzing the detected amounts of FB1 and FB2, differences between the applied treatments were observed. Namely, the analysis of the results shows that high amounts of FB1 and FB2 were detected in the control treatment and the synthetic fungicide treatment (T1 and T2), respectively. Significantly lower amounts of these two mycotoxins are detected in the T3 and T4 treatments, in which *T. harzianum* 179 was applied, while the lowest amount was detected in the T5 treatment. This was expected given that Yates et al. (1999) [61] and Rojo et al. (2007) [62] confirmed that *Trichoderma* suppresses the production of FB1.

However, an interesting result emerged in the detection of trichothecene mycotoxins. Namely, a significant amount of T-2 and HT-2 was detected in one of the samples treated with a synthetic fungicide (T2), while in all other samples these mycotoxins were not detected. Therefore, according to the obtained results, it can be concluded that the 2021 growing season was more favorable for the development of *Fusarium* than for *Aspergillus* species.

The results of mycotoxin analysis in samples from the 2022 growing season are shown in Table 4. The results of aflatoxin analysis show that the control samples (T1) contained significant amounts of aflatoxins B1 and B2, while the presence of these two mycotoxins was also observed in the synthetic fungicide treatment (T2) in slightly smaller amounts compared to the control. At the same time, aflatoxins G1 and G2 were not detected in samples. In contrast to these treatments, no aflatoxins were detected in the *T. harzianum* K179 treatments (T3, T4, and T5). Also, ochratoxin A was not found in any of the analyzed samples. These results are in accordance with the results of Ren et al. (2022) [63] and Dini et al. (2022) [64] who confirmed the positive effect of *Trichoderma* isolates in suppressing the production of aflatoxins and ochratoxin A.

By observing the detected amounts of ZEN and DON, it can be concluded that the presence of ZEN was observed only in the analyzed control samples. On the other hand, as in the previous 2021 season, the presence of FUM was observed in all tested samples. Also, the detected amounts of FUM are significantly higher than in the previous season. Analyzing the results, it can be noticed that during the 2022 season, the lowest FUM concentrations were observed in treatments T4 and T5. This is in accordance with the results shown in Figure 4, from which it can be noted that the treatments T4 and T5 had the highest number of healthy corn ears. Mycotoxins T-2 and HT-2 were not detected in any sample during the 2022 growing season.

Climate change, such as rising temperatures and changing rainfall patterns, has been found to be a key cause of fungal growth and crop contamination [65]. Considering that 2022 was a dry year compared to 2021, which was favorable for the multiplication of *Aspergillus* species [60], it was expected that aflatoxins would be present in the samples collected during the 2022 growing season. At the same time, the results showed that the use of *T. harzianum* K179 in biocontrol has great potential for suppressing aflatoxin-producing *Aspergillus* species.

## 4. Discussion

The results obtained in the in vitro biocontrol experiment shows a large difference in the sensitivity of two maize phytopathogens, *F. graminearum* K28 and *A. flavus* K328; however, it is considered that all diameters larger than 22 mm indicate that the applied agent is highly effective [66]. Thus, it can be concluded that the inhibition zone diameters obtained in the in vitro biocontrol experiment are significantly larger than the abovementioned value, and that *T. harzianum* K179 cultivation broth produced in the bioreactor under defined conditions can be used for the field experiment. These results match those of our previous research [49] and enabled us to conduct a complex trial in the field. Certainly, these results were expected given that the exceptional antifungal activity of *T. harzianum* against *A. flavus* was confirmed by Chiuraise et al. (2015) [67] and Kifle et al. (2017) [68] in their studies. Tian et al. (2018) [69], on the other hand, concluded in their study that three tested *Trichoderma* isolates, including the species *T. harzianum*, were proven to be potential candidates for control of *F. graminearum* ZEN producers.

By comparing the results obtained during the field trials carried out in 2021 and 2022 seasons, the certain differences were registered. Beneficial soil microbes, particularly those from the genus *Trichoderma*, are tested as viable and sustainable resources for promoting plant growth and alleviating biotic and abiotic stressors in research and commercial production environments [70]. Certainly, by analyzing the disease severity results, it can be concluded that during both growing seasons, the results showed exceptional efficacy of *T. harzianum* in controlling fungal infections of maize in the field. This is not surprising since the very successful seed treatment with *T. harzianum* on groundnut in controlling *A. flavus* and aflatoxins was confirmed by Chiuraise et al. (2015) [67]. Similarly, Saravanakumar et al. (2017) [71] in their study confirmed the exceptional potential of *T. harzianum* in controlling *F. graminearum* on maize. Results obtained in this research showed that it was necessary to either extend the treatment time or add an adjuvant (glue) in order to ensure that *T. harzianum* binds better to the seed and thus better exhibits its antifungal activity. Certainly, in addition to the pronounced impact on biotic stress and the significant use of *Trichoderma* spp. in biocontrol, this genus shows a remarkable impact on abiotic stress [72].

Based on Republic Hydrometeorological Service of Serbia (RHMS) [73] data on minimum and maximum temperatures and precipitation, it can be noticed that the 2022 season was extremely dry compared to the 2021 season. The significant differences in maize grain yield observed between the two studied years are probably the result of climatic factors, precipitation and air temperature, which are the most influential elements affecting maize cultivation in an observed region. Specifically, in 2022, when significantly lower grain yield was recorded, the observed site experienced the hottest summer since the beginning of meteorological measurements in 1951 (Seasonal Bulletin for Serbia, Republic Hydrometeorological Institute, 2022) [73]. In addition to extreme temperatures during the flowering and pollination phases, July—the most critical period for maize in terms of water needs, was characterized by only 13.8 mm of precipitation, which is almost ten times less than the maize water needs for that month. The combination of these unfavorable conditions led to the occurrence of extreme drought, which significantly reduced maize yields in the wider growing region. In contrast, in July 2021, the same site received 114.4 mm of precipitation, which proved to be a decisive factor contributing to the significant differences in yields between the two observed years (Supplementary Materials). Considering that climate change is a significant barrier to sustainable agricultural output in the twenty-first century, it can be assumed that probably the influence of abiotic stress is responsible for this difference. It is well known that alterations in precipitation patterns, carbon dioxide concentrations, temperature and the frequency and intensity of extreme events such as flooding, drought and hail significantly impede the attainment of food security for the growing population [74].

Given that the 2021 and 2022 seasons were completely different in terms of climatic conditions, we can conclude that the differences we obtained in this research for grain yield were expected. A variety of factors, including crop, genotype of the plant, biocontrol agent application method (soil, root and seed), delivery mechanism, inoculum concentration and environmental and soil conditions, might affect a plant’s reaction to *Trichoderma* spp. treatment. Our study provides valuable insights into how these different factors interact with *Trichoderma*, and our findings can guide future studies and practical applications by identifying the conditions under which *Trichoderma* is the most effective. Moreover, understanding how *Trichoderma* behaves under various conditions is crucial for optimizing its use in agriculture. Water deficiency stress triggers physiological reactions in plants, including reduced photosynthesis, stomatal closure, decreased cell development and increased respiration. Plants inoculated with *Trichoderma* improve water-deficit tolerance by regulating drought-induced changes in stomatal opening, enhancing root development, chlorophyll content in leaves and photosynthesis. Thus, using biological agents, such as water deficit-tolerant *Trichoderma*, could be a cost-effective, environmentally benign and long-term strategy for reducing drought stress [70].

Chepsergon et al. (2012) [75] verified the favorable influence of *T. hamatum* on the cocoa plant and *T. harzianum* T22 on tomatoes during drought. Also, Scudeletti et al. (2021) [76] tested *T. asperellum*’s impact on sugarcane during a drought. They found that the plant’s physiological characteristics, such as photosynthetic pigments, stomatal conductance, water use efficiency, and antioxidant metabolism, are all impacted by the microorganism’s inoculation. All of these elements contributed to plant growth and a high yield despite the drought. *T. harzianum* isolates PB23 and PB9 were also found to have a favorable effect on *Brassica juncea* plants under drought circumstances [77]. Cornejo-Ríos et al. (2021) [78] found that tomato plants primed with *T. asperellum* reduced physiological and agronomic symptoms associated with drought and chilling stress, and through improved antioxidant regulation and *T. koningii* showed helped provide tomato plants resistance to heat stress. When applied under high-temperature stress, this strain decreased the production of ROS (reactive oxygen species) and shielded the plant cells from oxidative damage [79].

The presence or absence of mycotoxins can be related to climatic conditions, but also to the presence/absence of adequate agents to control the fungi that produce them. During the 2021 growing season, it can be concluded that there were no detected mycotoxins produced by *Aspergillus* species. Given that warm, dry conditions favor the growth of *Aspergillus* species, it can be concluded that the 2021 season was probably not conducive to the growth of *Aspergillus* species and thus the production of aflatoxins and ochratoxin A [5,10]. What can also be noted is that the mycotoxins produced by *F. graminarum*, DON and ZEN, were also not detected during the 2021 season. On the other hand, presence of FB1 and FB2 proves that the maize was infected mainly by *F. verticillioides* or other *Fusarium* species from Liseola section. However, the amounts of these mycotoxins varied significantly among the applied treatments. Thus, the highest amount of detected fumonisins was present in the control sample, T1, while the lowest amount of FUM was present in treatments T4 and T5. These differences were expected given that Ferrigo et al. (2014) [57] confirmed that *T. harzianum* T22 induces in maize systemic resistance against *F. verticillioides*. Subsequently, Jambhulkar et al. (2022) [80] determined that *T. harzianum* BThr29 has the potential to be used in the control of *F. verticillioides* causing post flowering stalk rot in maize. Interestingly, T-2 and HT-2 were detected only in one sample treated with a synthetic fungicide.

During the 2022 growing season, the situation was different compared to the previous season. Namely, during this season, the presence of aflatoxins produced by *Aspergillus* species was detected in some samples. This was expected given that climatic conditions during the 2022 growing season were fully conducive to the growth of *Aspergillus* species. The results show that aflatoxins were detected in the T1 treatment in the highest amounts, as well as in the T2 treatment in a slightly smaller amount. However, it is interesting that aflatoxins were not detected in the treatments with the *T. harzianum* 179 cultivation broth. Considering that Chiuraise et al. (2015) [67], Kifle et al. (2017) [68] and Ren et al. (2022) [63] have demonstrated the very good antagonistic activity of *Trichoderma* isolates, especially *T. harzianum*, in the biocontrol of *Aspergillus* species, it can be concluded that the results obtained in this study were expected. Another difference compared to the 2021 growing season is the presence of ZEN mycotoxins in the control sample, which is an indicator of the presence of infection with the fungal species *F. graminearum*. However, Tian et al. (2018) [69] determined the antagonistic potential of *Trichoderma* spp. in the control of ZEN and *F. graminearum*. During the 2022 season, aflatoxins G1 and G2, ochratoxin A, DON, T-2, and HT-2 were not detected in the analyzed samples. However, as in the 2021 season, the presence of fumonisins B1 and B2 was registered in all samples during the 2022 season, but in significantly higher quantities.

So, by comparing the results for disease severity and the presence of mycotoxins, we can draw several conclusions. Notably, since the 2022 season was more favorable for fungal development and mycotoxin production (Table 4), the presence of mycotoxins was significantly higher compared to the 2021 season (Table 3). However, it is evident that all treatments involving *T. harzianum* 179 resulted in either the absence or a reduction of mycotoxin levels compared to the control treatment, and even compared to the synthetic fungicide treatment. At the same time, disease severity results for the 2022 season indicate that treatments T3–T5 were the most effective (Figure 4). Therefore, we can conclude that in the extreme conditions of 2022, *T. harzianum* 179 demonstrated strong biocontrol potential while also helping to mitigate the negative effects of abiotic stress. These findings further confirm the significant potential of the *Trichoderma* genus in alleviating both biotic and abiotic stress in plants [72].

## 5. Conclusions

This study shows the potential of sustainable solutions to some of agriculture’s biggest challenges. The fungus *T. harzianum* K179 proved to be an effective natural alternative to chemical fungicides for protecting maize from harmful fungal diseases and reducing toxic mycotoxin contamination. In field trials, seeds treated with this bioagent produced healthier plants, suffered less from disease, and had higher yields, even in seasons with challenging weather conditions. What is more, maize from treated plants contained significantly lower levels of harmful substances like aflatoxins and fumonisins, which are dangerous to both animals and humans. These results suggest that *T. harzianum* K179 not only enables better crop performance but also helps produce safer food and feed. This research paves the way for broader use of biocontrol agents in modern agriculture.

## Figures and Tables

**Table 1:** Parameter estimates of the proportional odds model for disease severity across different treatments (results for 2021).

Treatment	*β*	Lower CI	Upper CI	*p*
T2	−0.8710	−1.7423	−0.0246	0.046
T3	−3.8406	−4.9900	−2.7824	0.000
T4	−5.4154	−6.8135	−4.1615	0.000
T5	−4.5105	−5.7601	−3.3726	0.000

**Table 2:** Coefficient estimates of the proportional odds model for disease severity across different treatments (results for 2022).

Treatment	*β*	Lower CI	Upper CI	*p*
T2	−0.559	−1.404	0.274	0.201
T3	−1.908	−2.870	−0.993	0.000
T4	−3.402	−4.609	−2.302	0.000
T5	−2.543	−3.612	−1.550	0.000

**Table 3:** Presence of mycotoxins in maize samples from the 2021 growing season.

μg/kg	Aflatoxins				Ochratoxin A	ZEN	DON	Fumonisins		HT-2	**T-2**
	B1	B2	G1	G2				B1	B2		
M_I_ T1	<0.4	<0.4	<0.4	<0.4	<1.6	<16	<64	11,419	4384	<9.6	<9.6
M_II_ T1	<0.4	<0.4	<0.4	<0.4	<1.6	<16	<64	9037	3895	<9.6	<9.6
M_I_ T2	<0.4	<0.4	<0.4	<0.4	<1.6	<16	<64	10,848	3698	<9.6	<9.6
M_II_ T2	<0.4	<0.4	<0.4	<0.4	<1.6	<16	<64	3505	924	1478	692
M_I_ T3	<0.4	<0.4	<0.4	<0.4	<1.6	<16	<64	3822	1405	<9.6	<9.6
M_II_ T3	<0.4	<0.4	<0.4	<0.4	<1.6	<16	<64	2126	660	<9.6	<9.6
M_I_ T4	<0.4	<0.4	<0.4	<0.4	<1.6	<16	<64	2813	981	<9.6	<9.6
M_II_ T4	<0.4	<0.4	<0.4	<0.4	<1.6	<16	<64	2164	896	<9.6	<9.6
M_I_ T5	<0.4	<0.4	<0.4	<0.4	<1.6	<16	<64	1982	709	<9.6	<9.6
M_II_ T5	<0.4	<0.4	<0.4	<0.4	<1.6	<16	<64	1491	368	<9.6	<9.6

**Table 4:** Results of the presence of mycotoxins in maize samples from the 2022 growing season.

μg/kg	Aflatoxins				Ochratoxin A	ZEN	DON	Fumonisins		HT-2	T-2
	B1	B2	G1	G2				B1	B2		
M_I_ T1	2.9	0.6	<0.4	<0.4	<1.6	19	<64	6043	2704	<9.6	<9.6
M_II_ T1	3.2	0.8	<0.4	<0.4	<1.6	23	<64	25,867	12,569	<9.6	<9.6
M_I_ T2	0.8	0.5	<0.4	<0.4	<1.6	<16	<64	10,643	4552	<9.6	<9.6
M_II_ T2	0.6	0.4	<0.4	<0.4	<1.6	<16	<64	3664	1582	<9.6	<9.6
M_I_ T3	<0.4	<0.4	<0.4	<0.4	<1.6	<16	<64	7659	2780	<9.6	<9.6
M_II_ T3	<0.4	<0.4	<0.4	<0.4	<1.6	<16	<64	14,128	5790	<9.6	<9.6
M_I_ T4	<0.4	<0.4	<0.4	<0.4	<1.6	<16	<64	6526	2347	<9.6	<9.6
M_II_ T4	<0.4	<0.4	<0.4	<0.4	<1.6	<16	<64	6223	3028	<9.6	<9.6
M_I_ T5	<0.4	<0.4	<0.4	<0.4	<1.6	<16	<64	8765	4455	<9.6	<9.6
M_II_ T5	<0.4	<0.4	<0.4	<0.4	<1.6	<16	<64	5939	2423	<9.6	<9.6

## Data Availability

The original contributions presented in the study are included in the article and Supplementary Materials. Further inquiries can be directed to the corresponding author.

## Supplementary Materials

The following supporting information can be downloaded at: https://www.mdpi.com/article/10.3390/jof11060416/s1.
