# Supplementary figures and images for: Trichoderma harzianum in Biocontrol of Maize Fungal Diseases and Relevant Mycotoxins: From the Laboratory to the Field
Source: J Fungi (Basel). 2025 May 27;11(6):416. doi: 10.3390/jof11060416 (PMC12193939; doi:10.3390/jof11060416)

● 2021 ■ 2022

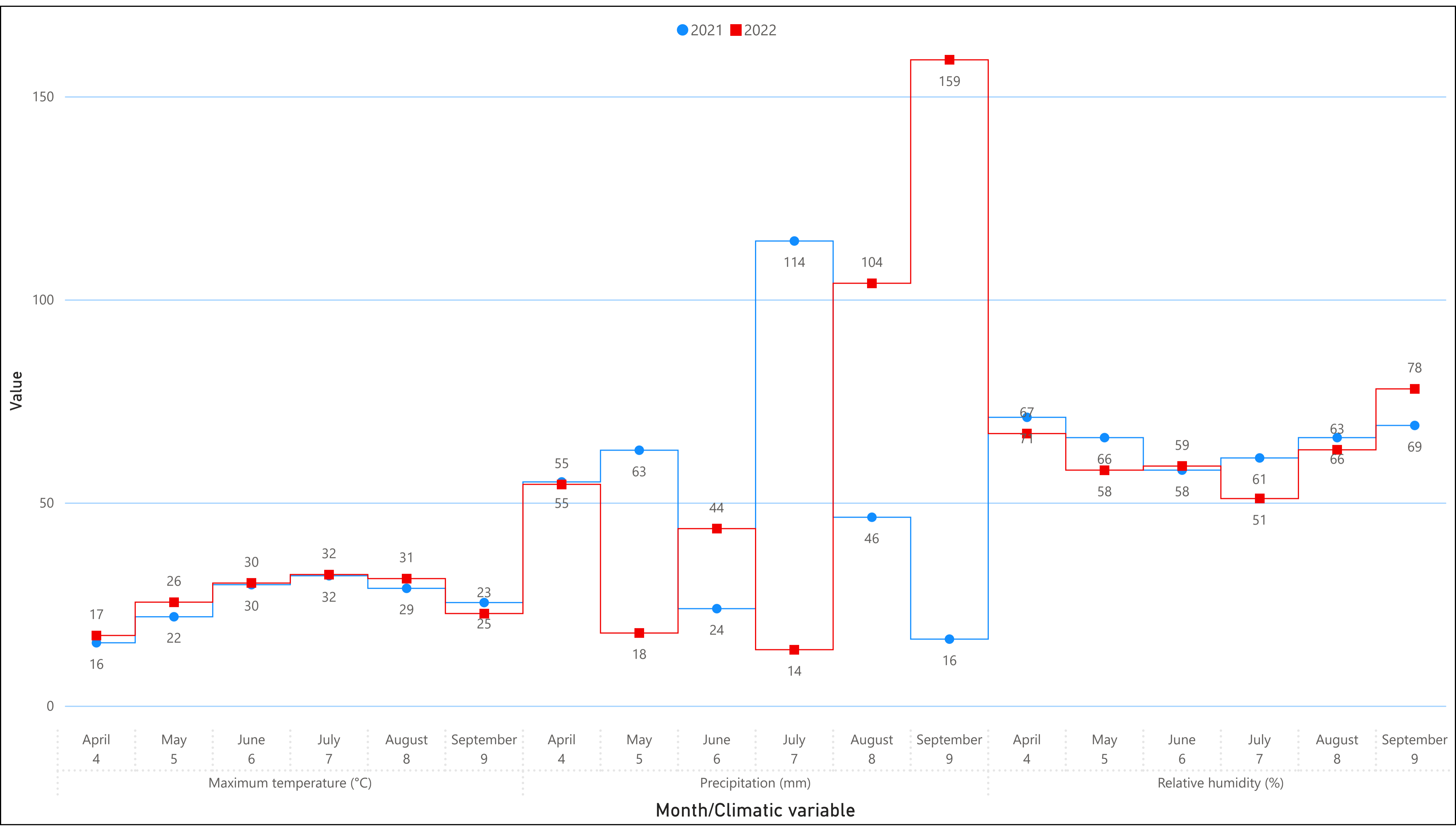

Supplement: Supplementary file 1 [file jof-11-00416-s001.zip › jof-3641960-supplementary.pdf]
